# Preoperative Diffusion-Weighted Imaging of Single Brain Metastases Correlates with Patient Survival Times

**DOI:** 10.1371/journal.pone.0055464

**Published:** 2013-02-05

**Authors:** Anna Sophie Berghoff, Thomas Spanberger, Aysegül Ilhan-Mutlu, Manuel Magerle, Markus Hutterer, Adelheid Woehrer, Monika Hackl, Georg Widhalm, Karin Dieckmann, Christine Marosi, Peter Birner, Daniela Prayer, Matthias Preusser

**Affiliations:** 1 Institute of Neurology, Medical University of Vienna, Vienna, Austria; 2 Department of Radiology, Division of Neuroradiology, Medical University of Vienna, Vienna, Austria; 3 Department of Medicine I, Medical University of Vienna, Vienna, Austria; 4 Department of Neurology, Wilhelm Sander NeuroOncology Therapy Unit, University Hospital Regensburg, Regensburg, Germany; 5 Austrian National Cancer Registry, Statistics Austria, Vienna, Austria; 6 Department of Neurosurgery, Medical University of Vienna, Vienna, Austria; 7 Department of Radiotherapy, Medical University of Vienna, Vienna, Austria; 8 Clinical Institute of Clinical Pathology, Medical University of Vienna, Vienna, Austria; 9 Comprehensive Cancer Center CNS Tumors Unit, Medical University of Vienna, Vienna, Austria; University of Maryland, College Park, United States of America

## Abstract

**Background:**

MRI-based diffusion-weighted imaging (DWI) visualizes the local differences in water diffusion *in vivo*. The prognostic value of DWI signal intensities on the source images and apparent diffusion coefficient (ADC) maps respectively has not yet been studied in brain metastases (BM).

**Methods:**

We included into this retrospective analysis all patients operated for single BM at our institution between 2002 and 2010, in whom presurgical DWI and BM tissue samples were available. We recorded relevant clinical data, assessed DWI signal intensity and apparent diffusion coefficient (ADC) values and performed histopathological analysis of BM tissues. Statistical analyses including uni- and multivariate survival analyses were performed.

**Results:**

65 patients (34 female, 31 male) with a median overall survival time (OS) of 15 months (range 0–99 months) were available for this study. 19 (29.2%) patients presented with hyper-, 3 (4.6%) with iso-, and 43 (66.2%) with hypointense DWI. ADCmean values could be determined in 32 (49.2%) patients, ranged from 456.4 to 1691.8*10^−6 ^mm^2^/s (median 969.5) and showed a highly significant correlation with DWI signal intensity. DWI hyperintensity correlated significantly with high amount of interstitial reticulin deposition. In univariate analysis, patients with hyperintense DWI (5 months) and low ADCmean values (7 months) had significantly worse OS than patients with iso/hypointense DWI (16 months) and high ADCmean values (30 months), respectively. In multivariate survival analysis, high ADCmean values retained independent statistical significance.

**Conclusions:**

Preoperative DWI findings strongly and independently correlate with OS in patients operated for single BM and are related to interstitial fibrosis. Inclusion of DWI parameters into established risk stratification scores for BM patients should be considered.

## Introduction

Metastases to the brain are a frequent complication of cancer and are associated with high morbidity and mortality. Primary tumor types vary in their propensity to form brain metastases (BM) with lung cancer, breast cancer and melanoma showing the highest incidences of central nervous system (CNS) involvement. [1], [2] Treatment so far relies mainly on surgery and radiotherapy, although some targeted drugs have shown clinically meaningful activity in distinct molecular tumor subtypes and are beginning to enter clinical practice. [3], [4], [5].

The prognosis of BM patients is poor with median overall survival times of only few months. Several risk stratification scores have been developed such as the recursive portioning analysis (RPA), the graded prognostic assessment (GPA) and the diagnosis specific graded prognostic assessment (DS-GPA). [6], [7], [8] These scores are based on parameters with established prognostic impact including the Karnofsky performance status (KPS), patient age, status of the primary tumor, presence of extracranial metastases and the number of BM. [6], [7], [8] Median overall survival (OS) from diagnosis of BM varies extensively from 3 months in the least favourable group, up to 25.3 months in the most favourable groups which includes also long term survivors. [8], [9] Neuroradiological variables, with the exception of the number of BM, are not considered for prognostic risk stratification so far.

Magnetic Resonance Imaging (MRI) using pre- and post-contrast T1-weighted imaging, T2-weighted imaging and fluid attenuated inversion recovery (FLAIR) is the modality of choice for radiological evaluation of brain tumors. [10] Increasingly, additional advanced radiological techniques like magnetic resonance spectroscopy (MRS), perfusion MRI, or diffusion-weighted imaging (DWI) are used to characterize brain lesions in order to provide further clinically relevant information. [11], [12] DWI is an MRI method based on the visualisation of the mobility of water molecules in the extracellular space. A low diffusion capacity due to a restricted mobility of the water molecules in the extracellular space results in a hyperintense signal in DWI and low apparent diffusion coefficient (ADC) values. In contrast, a high diffusion capacity due to an increased mobility of water molecules results in a hypo- or isointense DWI signals and high ADC values. [12] DWI parameters have been shown to correlate with various histopathological characteristics such as tumor type, tumor grade, Ki67 tumor cell proliferation index, cellularity, or amount of interstitial fibrosis and survival prognosis in several intra- and extracranial tumor types. [13], [14], [15], [16], [17], [18], [19], [20], [21]. However, the prognostic value of DWI and its correlation with histomorphological findings in patients with BM has not been systematically studied so far.

In the present study, we investigated the prognostic impact of DWI signal intensity and performed a correlative analysis with tissue-based parameters in a homogenous cohort of patients with single BM and surgery as first line treatment for BM.

## Patients and Methods

### Ethics Statement

The study was approved by the local ethics committee of the Medical University of Vienna, Austria. No written consent was given by the patients for their information to be stored in the database and used for research, because this study was performed in a retrospective manner in line with local regulations. The institutional ethics committee waived the need for written informed consent from the participants for this project (Ethics committee protocol number 641/2011).

### Patients

We identified all patients with radiologically proven single BM who underwent surgery as a first-line-therapy for a single BM between April 2002 and December 2010 and whose presurgical MRI work-up included DWI. Availability of at least one tissue block for research purposes with viable BM tissue and full information on the clinical course including date of diagnosis, administered therapies, Karnofsky performance score, GPA and date of death or date of last follow-up investigation were mandatory for inclusion. All clinical parameters were retrieved by chart review and from the database of National Cancer Registry of Austria and the Austrian Brain Tumor Registry. [22].

### Imaging Analysis

All imaging analyses were performed by one investigator (TS) blinded to all clinical and histological data. In conventional MRI (contrast-enhanced and native T1-weighted images, FLAIR and T2-weighted images, as available) the maximum diameter and localization of the single BM was determined. In DWI, the BM was semiquantitatively judged to be either hypointense, isointense, or hyperintense in comparison to normal non-pathological brain tissue. In BM which showed heterogeneous signal behaviour, diffusion intensity was graduated upon the predominant (>70% of the metastasis) signal behaviour. In the cases with available ADC maps, ADC values were derived as described previously in up to 5 non-overlapping areas of interest (each at least 50 mm2) in solid, non-necrotic, non-macrohemorrhagic areas of the BM. In each case, the mean ADC value (ADCmean) was calculated from the ADC values of all areas of interest. [13].

### Tissue Based Analysis

Tissue based analysis was performed blinded to clinical and radiological data. Histological confirmation of BM was evaluated on routinely performed hematoxylin and eosin (H&E) stained tissue sections by a specialist in neuropathology. Cellularity was evaluated semiquantatively on an H&E section as low, moderate and high. Gomori silver impregnation stain for reticulin was performed according to laboratory standard. The amount of extracellular reticulin fibers was semiquantitatively grouped as follows: prominent interstitial fibrosis (more than 25% of the tumor tissue displaying a dense interstitial meshwork of reticulin fibres); little interstitial fibrosis (less than 25% of the tumor tissue displaying a dense interstitial meshwork of reticulin fibres). Ki67 (antibody MIB1, Dako, Glostrup, Denmark) immunostaining and analysis was performed as previously published. [23] Ki67 proliferation index was obtained by counting 500 cells and giving the percentage of positive cells (0–100%). [23] Differentiation of the tumor tissue was divided into well, moderately and poorly differentiated based on the tumor organization, the cell polymorphism and the mitotic activity.

### Statistical Analysis

For correlation of parameters the Spearmańs correlation coefficient, Chi square test or the Mann-Whitney U test were used as appropriate. Overall survival (OS) was defined as time from first diagnosis of BM until death or last day of follow up. For all tests, a two-sided p-value of <0.05 was considered as statistically significant. For univariate survival analysis Kaplan-Meier curves and the log-rank test were used. Variables that showed statistically significant prognostic value at univariate survival analysis were entered in multivariate survival analysis using the Cox regression model.

The statistical software package SPSS version 19 (SPSS Inc, Chicago, IL, USA) was used for all calculations.

## Results

### Patients’ Characteristics

65 patients (31 female, 34 male) with a median age of 59 years (range 33–80) at first diagnosis of BM were available for this study. All patients had a single BM and surgery as first line treatment for BM. 45/65 (69.2%) of patients had adjuvant whole brain radiotherapy (WBRT) and 28/65 (43.1%) adjuvant chemotherapy after surgery of BM. **Table 1** lists further patients’ characteristics.

**Table 1:** Patients’ characteristics.

Parameter	Patient population (n = 65)	
	n	%
Age at first diagnosis of BM, years, (range)	59 (33–80)	
Primary tumor type		
Lung cancer	25	38.5
Breast cancer	8	12.3
Melanoma	5	7.7
Kidney cancer	5	7.7
Colorectal cancer	3	4.6
Others	19	29.2
Synchronous diagnosis of BM and primary tumor		
yes	29	44.6
no	36	55.4
Status of primary tumor at first diagnosis of BM		
No evidence of disease	19	52.8
Stable disease	11	30.6
Progressive disease	6	16.7
Presence of extracranical metastases		
yes	43	66.2
no	22	33.8
Karnofsky performance score		
<70	4	6.2
>70	61	93.8
GPA class		
I	23	35.4
II	15	23.1
III	25	38.5
IV	2	3.1
Number of BM		
Single BM	65	100
BM localisation		
Infratenorial	19	29.2
Supratentorial	46	70.8
Size of BM		
<3 cm	22	33.8
>3 cm	43	66.2
DWI signal intensity		
Hypointense	43	66.2
Isointense	3	4.6
Hyperintense	19	29.2
ADC_mean_ (range)	969.47*10^−6^ mm^2^/s (456.38–1691.80)	
1^st^ line treatment for BM		
Surgery	65	100
WBRT after surgery	45	69.2
Chemotherapy after surgery	28	43.1
OS from first diagnosis of BM, months (range)	15 (0–99)	

### Imaging Analysis

19/65 (29.2%) of patients presented with hyperintense, 3/65 (4.6%) with isointense and 43/65 (66.2%) with hypointense DWI signals. Clinical characteristics including primary tumor types, size of BM, patient age, status of primary tumor, presence of extracranial metastases and GPA did not differ between DWI signal intensity groups (p>0.05; Chi square test; **table 2**).

**Table 2:** Diffusion weighted imaging analysis.

Parameter	DWI signal intensity				Chi square	
	Hypo/isointense		Hyperintense			
	n	%	n	%		
Age at first diagnosis of BM						
<60 years	26	74.3	9	25.7	0.50	
>60 years	20	66.7	10	33.3		
Primary tumor type						
Lung cancer	19	76	6	24	0.22	
Breast cancer	6	75	2	35		
Melanoma	3	60	2	40		
Kidney cancer	5	100	0	0		
Colorectal cancer	3	100	0	0		
Others	10	53.8	9	46.2		
Status of primary tumor at first diagnosis of BM						
Synchronous diagnosis	20	69	9	31	0.14	
No evidence of disease	14	73.7	5	26.3		
Stable disease	7	63.6	4	36.4		
Progressive disease	5	83.3	1	16.7		
Presence of extracranial metastases						
yes	13	59.1	9	40.9	0.16	
no	33	76.7	10	23.3		
Karnofsky performance score						
<70	1	25	3	75	0.04	
>70	45	73.8	16	26.2		
GPA class						
I	19	82.6	4	17.4	0.45	
II	10	66.7	5	33.3		
III	16	64	9	36		
IV	1	50	1	50		
BM localisation						
Infratentorial	14	73.7	5	26.3	0.74	
Supratentorial	32	69.6	14	30.4		
Size of BM						
<3 cm	16	72.7	6	27.3	0.80	
>3 cm	30	69.8	13	30.2		
ADC_mean_						
<969.47*10^−6^ mm^2^/s	8	44.4	10	55.6	0.001	
>969.47*10^−6^ mm^2^/s	15	100	0	0		
Celluarity						
Low	9	64.3	5	35.7	0.86	
Moderate	22	73.3	8	26.7		
High	15	71.4	6	28.6		
Differentiation						
Low	34	73.9	12	26.1	0.50	
Moderate	8	66.7	4	33.3		
High	3	100	0	0		
Ki67 proliferation index						
0–25%	12	75	4	25	0.81	
25.1–50%	15	71.4	6	28.6		
50.1–75%	17	70.8	7	29.2		
75.1–100%	2	50	2	50		
Interstitial fibrosis						
Little	33	80.5	8	19.9	0.02	
Prominent	13	54.2	11	45.8		

ADC maps were available for 32 patients. The median ADCmean value was 969.47*10^−6 ^mm^2^/s. ADC values strongly correlated with signal intensity in isotropic DWI (p<0.001, Mann-Whitney U test; **table 2**). Clinical characteristics including primary tumor types, size of BM, patient age, status of primary tumor, presence of extracranial metastases, GPA and KPS did not correlate with ADCmean values (p>0.05; Mann Whitney U test).

### Tissue Based Findings

14/65 (21.5%) specimens were classified with low, 30/65 (46.2%) with moderate and 21/65 (32.3%) with high cellularity based on H&E histomorphology.

**Figure 1:**
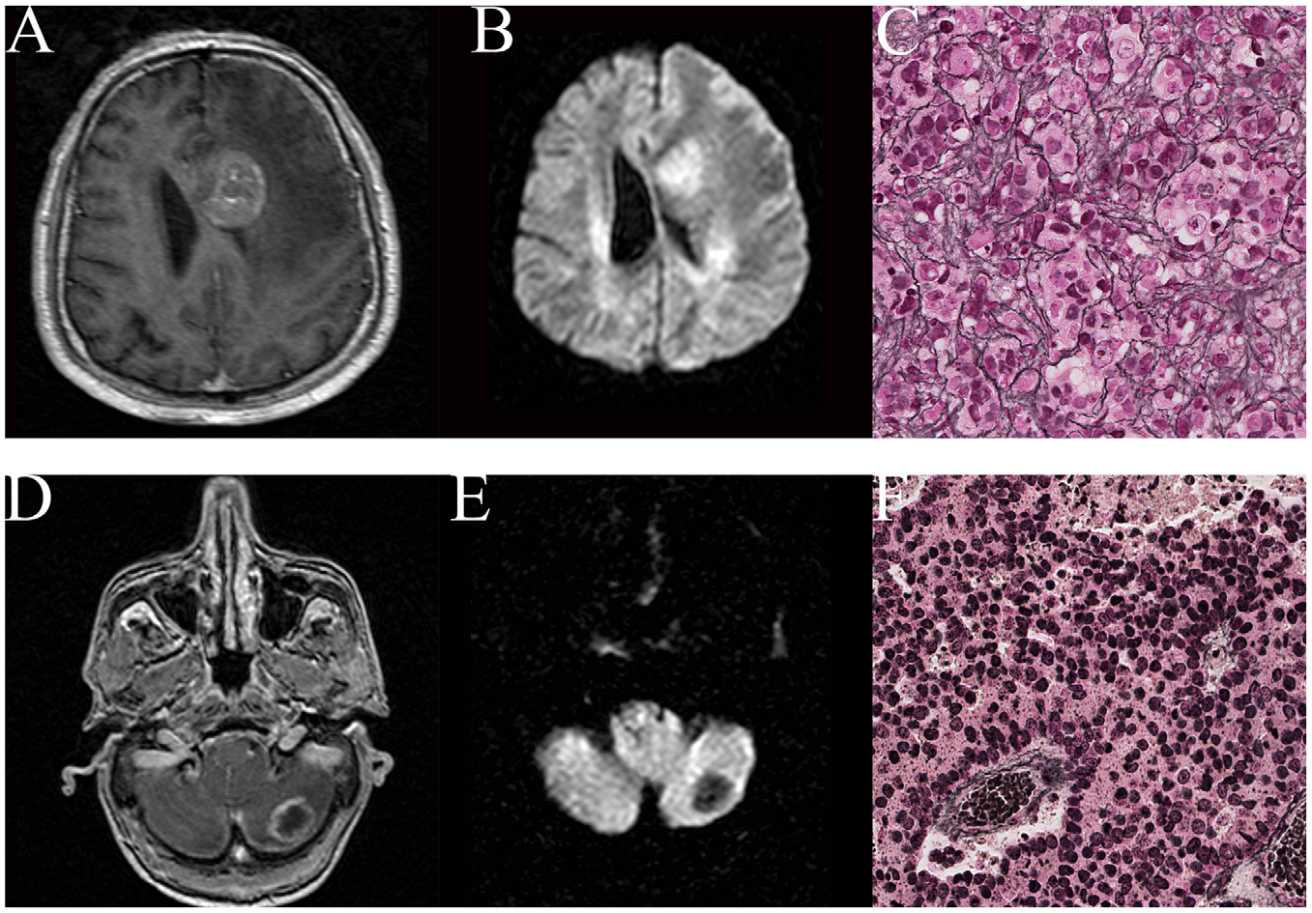
T1-weighted and diffusion weighted imaging of a patient with hyperintense DWI signal intensity (A, B) and of a patient with hypointense DWI signal intensity (D, E) and the Gomori silver impregnation stain for reticulin in these patients showing dense reticulin network (C) and scattered reticulin network (F).

No statistically significant correlation of DWI signal intensity or ADCmean and cellularity was observed (p>0.05; Chi square test and Mann-Whitney U test, respectively). 3/65 (4.6%) specimens were classified as well differentiated, 12/65 (18.5%) as moderately differentiated and 46/65 (70.8%) as poorly differentiated. No statistically significant correlation of DWI signal intensity or ADCmean and differentiation was observed (p>0.05; Chi square test and Mann-Whitney U test, respectively). Mean ki67 proliferation index was 44.4% (range 5.4% –89.6%) and did not correlate with DWI signal intensity (p>0.05; Mann-Whitney U test) or ADCmean values (Spearmańs correlation coefficient r =  - 0.3, p = 0.09). 24/65 (36.9%) specimens presented with prominent interstitial fibrosis while 41/65 (63.1%) showed little interstitial fibrosis. Semiquantitative DWI signal intensity showed a significant correlation with density of the reticulin network: tumors with restricted diffusion showed higher amounts of interstitial fibrosis and tumors with unrestricted diffusion showed less interstitial fibrosis (p = 0.02; Chi square test; **table 2** **,** **figure 1**).

### Survival Analyses

Median OS from first diagnosis of BM to death was 15 months (range 0–99 months) in the entire population.

**Figure 2:**
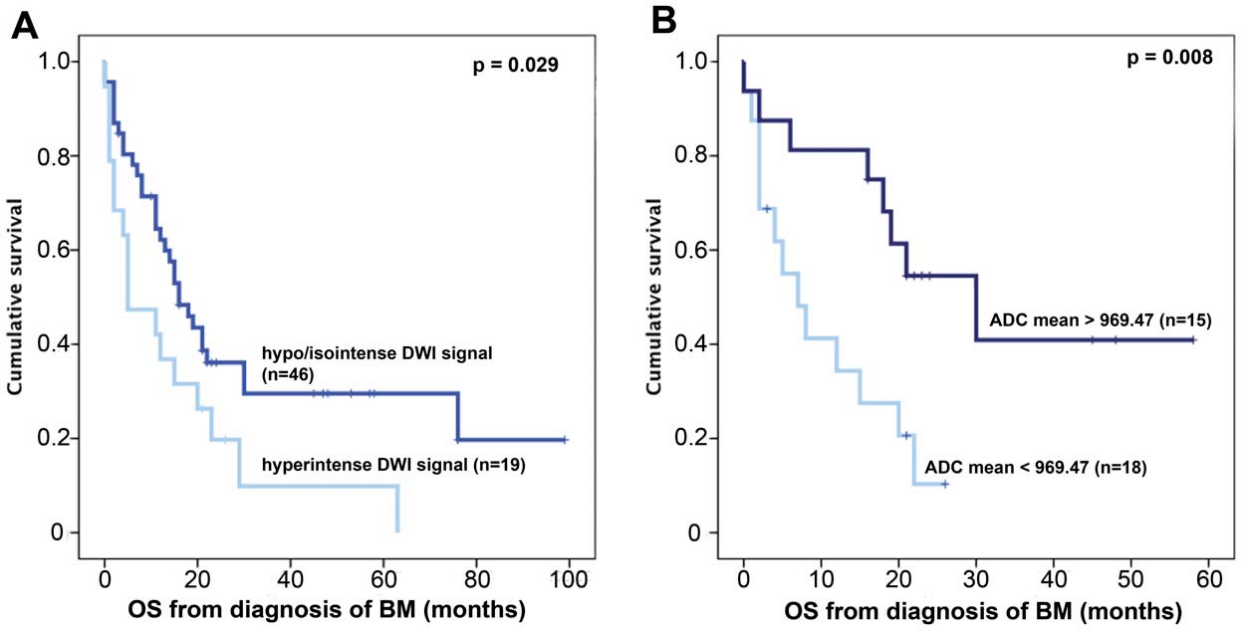
Kaplan Meier plots showing the statistically significant association of DWI signal intensity (A), ADC_mean_ values (B).

In univariate analysis, patients with hypo/isointense DWI signal intensity showed a significantly longer survival with a median OS of 16 months (95% CI: 10.79–21.25) than patients with hyperintense DWI signal intensity with a median OS of 5 months (95% CI: 0–12.47; p = 0.029; log rank test; **figure 2**). Patients with high ADCmean values showed a significantly longer survival with a median OS of 30 months (95% CI: 13.97–46.03) than patients with low ADCmean values with a median OS of 7 months (95% CI: 1.51–12.49; p = 0.02; log rank test; **figure 2**). Furthermore, primary tumor type, Karnofsky performance score <70, lack of adjuvant WBRT after neurosurgery and high GPA were significantly associated with unfavourable OS in univariate analysis (p<0.05). Adjuvant chemotherapy after surgery for BM had no statistically significant impact on OS (p>0.05).

All factors with statistically significant impact on OS in the univariate analysis were included in multivariate analysis (ADCmean, primary tumor type, KPS and adjuvant WBRT (yes/no)). Primary tumor type as well as KPS and adjuvant WBRT did not remain statistically significant in multivariate analysis. Only high ADCmean values remained as statistically significant independent prognostic factors (Harzard ratio 0.32, 95% CI 0.12–0.91; p = 0.03; Cox regression model; **table 3**).

**Table 3:** Survival analysis from first diagnosis of brain metastasis to death.

Parameter	Median OS, months	95% Confidence interval	Log-rank test	Cox regression model
ADC_mean_				
<969.47	7	1.51–12.49	0.008	0.03
>969.47	30	13.97–46.03		
Primary tumor type				
Lung cancer	21	11.81–30.19	0.015	0.64
Breast cancer	12	0–31.40		
Melanoma	4	0.78–7.22		
Kidney cancer	not reached	not reached		
Colorectal cancer	11	0–22.20		
Others	12	5.76–18.24		
Karnofsky performance				
<70	1	0–3.94	0.001	0.06
>70	15	11.73–24.27		
GPA class				
I	21	16.57–25.43	0.001	0.48
II	21	1.77–40.23		
III	12	0.28–23.73		
IV	0	-		
WBRT after surgery				
Yes	18	12.66–23.34	0.034	0.41
No	5	0–11.57		

## Discussion

In this study, we found a highly significant association of pre-surgical DWI parameters with OS times of patients operated for single BM. Both, DWI signal intensity as assessed semiquantitatively by visual impression and ADCmean values stratified patients into prognostic groups. The median OS of patients with tumors showing hyperintense DWI was 5 months compared to 16 months in patients with iso- or hypointense DWI signals (p = 0.029; log rank test). ADCmean values showed an even stronger separation of risk groups with patients with high ADCmean values showing more than 4-times longer median OS (30 months) than patients with low ADCmean values (7 months; p = 0.008; log rank test). The prognostic impact of ADC values was independent from known prognostic factors including GPA class, the primary tumor type and the KPS and also from postoperative therapy including adjuvant WBRT and chemotherapy in multivariate analysis (Hazard ratio 0.32, 95% CI 0.12–0.91; p = 0.03; Cox regression model).

While, to the best of our knowledge, the correlation of DWI parameters with patient outcomes has not been investigated in BM, some studies have postulated a prognostic value of DWI signal intensity in primary tumors. In colorectal cancer, low mean ADC values were shown to be associated with aggressive tumor behaviour, impaired response to therapy and decreased prognosis. [19] Similarly, a correlation of low ADC values and impaired OS and impaired progression free survival was postulated for several other extracranial cancers with high propensity to form BM like lung cancer or breast cancer. [21], [24] For the instance of intracranial lesions, hyperintense DWI signal were shown to be associated with worse outcome in primary CNS lymphoma and pituitary macroadenoma. [16], [25] In line with these findings we observed a statistically significant and independent adverse prognostic value of restricted diffusion in our homogenous cohort of patients with single BM. Therefore, DWI signal intensity could serve as a prognostic imaging biomarker for clinical decision making.

The DWI signal intensity was shown to correlate with histology and cellularity of various intra- and extracranial tumors, providing an indirect insight in a tumor’s microarchitecture. [13], [15], [19], [21], [26] In high grade gliomas a hyperintense DWI signal with low ADCmean values resembles areas of high cellularity with high cytoplasm to nucleus ratio. [14], [15] Similar, a correlation of hyperintense DWI signal intensity and poor tumor differentiation was shown for extracranial tumors like lung cancer, breast cancer or rectal cancer. [19], [21], [24] For the instance of BM, a low ADCmean value was shown to correlate with high tumor cellularity and poor tumor differentiation. [26], [27] In our study, we could demonstrate a significant correlation of a prominent interstitial fibrosis with signs of restricted diffusion in DWI, which resembles the impaired mobility of water molecules in the intercellular space. The interstitial reticulin fiber network is part of the fibrotic collagen-rich tumor stroma and our data further emphasize the importance of the microenvironment in the pathobiology of BM. [28] In line with our results, several other tumor types with a restricted diffusion due to dense stromal matrix were shown to have an impaired survival prognosis. [29], [30], [31].

Our study has some limitations that need to be acknowledged. We performed a retrospective study in a single center and were therefore able to include only a limited number of cases, thus restricting the statistical power of our correlative analyses. On the other hand, our approach enabled us to analyse a well-defined patient cohort characterized by single brain metastasis homogenously treated by neurosurgical BM resection as the initial therapy. Another limitation related to the retrospective nature of our study is the fact that ADC maps could not be retrieved in all cases. In the absence of ADC maps, diffusion restriction as cause of DWI hyperintensity cannot be unequivocally differentiated from other phenomena such as T2-shine through. [32] Further, the accuracy of ADC values is potentially limted due to the usage of different MRI machines. [33] However, we found a strong correlation of ADC values and semiquantitaitve DWI signal intensity in the cohort of 32 patients of whom both parameters were available. Furthermore, in this cohort the prognostic impact of ADC values was even more pronounced than the semiquantitatively evaluated DWI signal intensity. Still, our findings need to be reproduced in independent data sets, preferably in prospective studies.

In conclusion, we could demonstrate the independent prognostic value of DWI findings in our large homogenous cohort of patients with a single BM and its correlation with tissue based characteristic, indicating the value of DWI signal intensity as an imaging biomarker. Future studies should prospectively evaluate the prognostic value and the inclusion in prognostic scores of DWI parameters.
